# The Role of the Innate Immune System in Granulomatous Disorders

**DOI:** 10.3389/fimmu.2013.00120

**Published:** 2013-05-24

**Authors:** Helen J. Petersen, Andrew M. Smith

**Affiliations:** ^1^Eastman Dental Institute, University College LondonLondon, UK; ^2^Department of Medicine, University College LondonLondon, UK

**Keywords:** granuloma, Crohn’s, innate, macrophage, neutrophil, autophagy

## Abstract

The dynamic structure of the granuloma serves to protect the body from microbiological challenge. This organized aggregate of immune cells seeks to contain this challenge and protect against dissemination, giving host immune cells a chance to eradicate the threat. A number of systemic diseases are characterized by this specialized inflammatory process and granulomas have been shown to develop at multiple body sites and in various tissues. Central to this process is the macrophage and the arms of the innate immune response. This review seeks to explore how the innate immune response drives this inflammatory process in a contrast of diseases, particularly those with a component of immunodeficiency. By understanding the genes and inflammatory mechanisms behind this specialized immune response, will guide research in the development of novel therapeutics to combat granulomatous diseases.

## Introduction

The innate immune system plays a central role in driving inflammation and protecting the body from invading microorganisms. This first line of defense is made up of white blood cells, secretory molecules, and proteins which form an anatomical barrier to infection. Components of the innate immune system are important for orchestrating granuloma formation in response to microbial and foreign body challenge. This specialized inflammatory process can occur throughout the body, varying in size, organization, and composition of innate immune cells involved. The ultimate function of the granuloma is to contain and eradicate microorganisms or chronic irritants that are resistant to elimination through the normal immune response. This process is finely balanced between host protection and localized collateral damage to normal tissue and potential loss of function.

An emerging concept that this specialized inflammatory process is in fact a last resort when other immune protective processes have failed is slowly becoming recognized. A number of immunodeficiency disorders hereditary and acquired, present with granulomatous inflammation at a variety of body sites. Granuloma formation in patients with these disorders often has an infective prequel. Failure to clear the infective organism results in persistence and subsequent granuloma formation to try to contain the infection. The aim of this review is to explore the role of the innate immune system and its aberrant disorders in the formation of the granuloma.

## Granuloma Formation and Role in Immunodefense

A granuloma is a collection of inflammatory cells, predominantly mature macrophages that form an aggregate in response to an antigen. This antigen can include invading bacteria, fungi, foreign bodies, and immune complexes (James, 2000; Schappi et al., 2008; Ramakrishnan, 2012). The purpose of the granuloma is to isolate this antigen from the body and to facilitate its eradication. This crucial immune reaction serves to protect the body from dissemination of the antigen, this being especially important in the case of mycobacterial infections. Immune defects, in particular ones that specifically affect the innate immune system have been shown to result in poor granuloma formation. Deficiency in tumor necrosing factor alpha (TNFα), Interleukin 12 (IL-12), or interferon gamma (IFNγ) also result in poor granuloma formation (Ramakrishnan, 2012).

**Figure 1:**
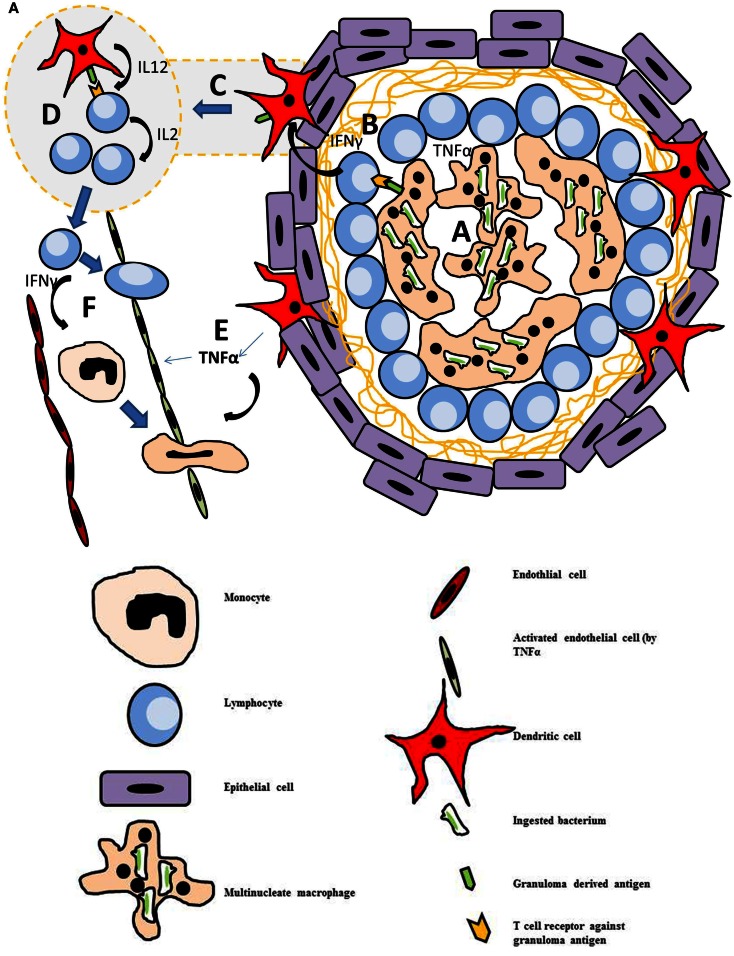

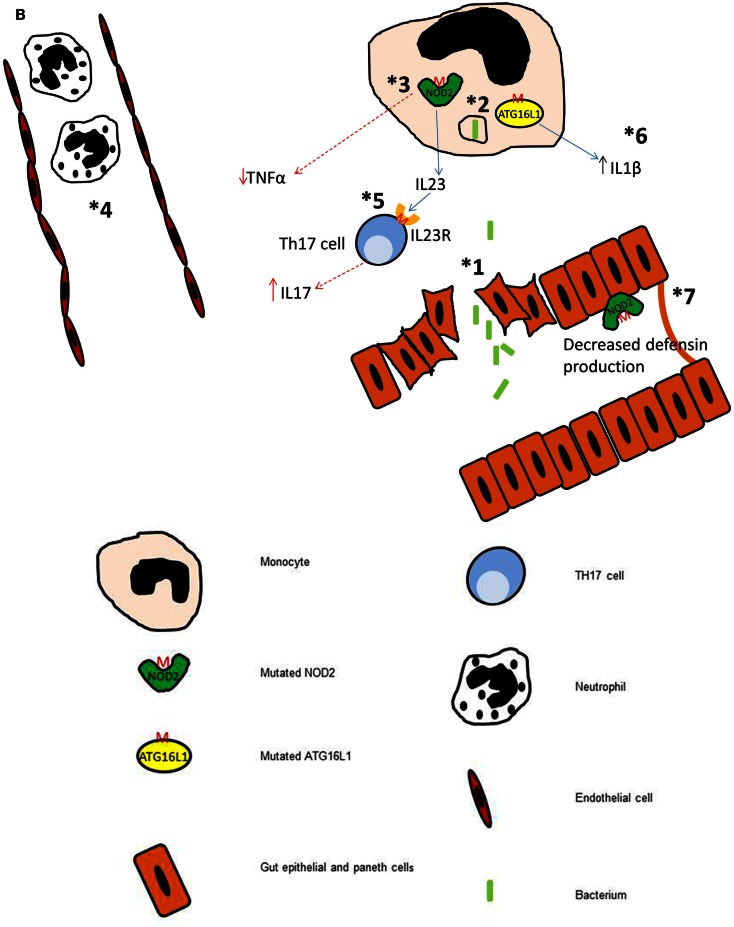
**(A)** Diagram of a mature granuloma. After 3 weeks of maturation, the granuloma is fully formed. Crosstalk between various cells of the immune system leads to proliferation of lymphocytes, predominantly T-helper cells. (a) Macrophages engulf the antigen and secrete pro-inflammatory cytokines. They also present antigen derived peptides and lipids via MHC class II and CD 1 molecules to T cells, natural killer T cells (NKT cells), and natural killer cells (NK cells). (b) Thl cells secrete IFNγ which activates dendritic cells. (c) Dendritic cells loaded with antigen migrate to the local lymph nodes where they present to naive CD4+ T cells. (d) Within the lymph node, the dendritic cells secrete IL-12 which stimulates these naive cells to differentiate into Thl cells. These in turn secrete IL2 to expand their population. (e) At the granuloma site, activated dendritic cells secrete copious amounts of TNFα which activates the endothelium, upregulating the number of adhesion molecules to allow extravasation of Thl cells and monocytes. (f) Thl cells secrete IFNγ which stimulates monocytes to differentiate into macrophages. **(B)** Diagrammatic representation of the multistep pathogenesis of Crohn’s disease. (1) Loss of integrity in the gut epithelium allows bacteria to leave the lumen and enter the tissue of the gut. (2) Defective autophagy. (3) Mutation of NOD2 leads to reduction in secretion of pro-inflammatory cytokines including TNFα. (4) Defect in Neutrophil function (monogenic diseases) or reduced chemotaxis (inherited or due to reduced TNFα secretion). This leads to persistence of bacterium in the tissues. (5) Mutational the IL23R on Thl7 cells leads to increased survival of these cells and increased IL17 production. High levels of IL17 is associated with increased bowel inflammation in Crohn’s. (6) Mutation of ATG16L (T300A) leads to a excessive of production of IL-lβ. (7) Mutation in NOD2 leads to reduced defensin production by intestinal Paneth cells.

On initiation of the granulomatous response, antigen presenting cells encountering an antigen release a cocktail of pro-inflammatory cytokines and chemoattractants. This recruits neutrophils from the circulation into the infected area and these in turn release additional cytokines to attract and activate monocytes. Under normal circumstances the recruited neutrophils eliminate the infective agent through the process of phagocytosis and digestion within the phagocytic vacuole (Segal, 2005). When the infective agent is resistant to neutrophil clearance macrophages engulf the antigen. Upon internalization, macrophages secrete additional pro-inflammatory mediators and attempt to digest the foreign body in order to present antigen derived peptides and lipids via MHC class II and CD1 molecules to T cells, natural killer T cells (NKT cells), and natural killer cells (NK cells). IFNγ secreted by NK cells, NKT cells, and T cells, activates dendritic cells. The dendritic cells in turn release copious amounts of TNFα to further promote the influx of immune cells to the area, creating a specialized microenvironment to deal with the foreign antigen (Figure 1A). Upon internalization of the antigen, macrophages secrete additional pro-inflammatory mediators and attempt to digest the foreign body in order to present antigen derived peptides and lipids via MHC class II and CD1 molecules to T antigen. The dendritic cells also migrate to the local lymph nodes to present the antigen to naïve CD4 cells. On secretion of IL-12 by dendritic cells, these naïve cells differentiate into T-helper 1 (Th1) cells, which secrete IL-2 to promote their survival and expand their population. Activated Th1 cells enter the circulation and are attracted into the granuloma through the high levels of chemokines and adhesion molecules present on the macrovascular endothelial cells. They move about the granuloma using the macrophages as a scaffold to crawl over (Egen et al., 2008), the granuloma resembles a fluid bag rather than a “walled off” solid structure with cells freely moving within in it (Savill and Fadok, 2000; Taylor et al., 2008).

Persistence of the antigen past this point leads to further chronicity of the inflammation and a mature granuloma. Complex cross talk between the individual immune cells and the surrounding epithelium via the production of high levels of TNFα and IFNγ leads to further maturation of macrophages. These macrophages become multinucleate giant cells, with increased phagocytosis and bacterial digestion capabilities. This is all aimed at trying to eradicate the antigen. After a number of weeks, these cells become terminally differentiated into epithelioid macrophages which contain large number of lysosomes, mitochondria, and are engaged in continued endocytic activity. Well-developed pseudopodia, allows for close interdigitation and a tightly packed aggregation of these enlarged cells. Finally, fibrosis occurs.

### Relation to human disease

A plethora of diseases in humans feature granulomatous inflammation including infective, neoplastic, vasculitic immunodeficiency states, and inflammopathies (Table 1). The rate of turnover of macrophages is key to the histological presentation of the granuloma. A highly toxic stimulus will result in macrophage death, such as seen in epithelioid granulomas. Examples of this are granulomas seen in tuberculosis and cat’s scratch disease. This results in constant influx of new macrophages to replace the dead cells. Whereas, an inert stimulus will result in a granuloma with low turnover of macrophages.

**Table 1:** **Table of granulomatous disorders**.

Granulomatous disease
**I. INFECTIONS**
• Fungi
○ *Histoplasma*
○ *Coccidioides*
○ *Blastomyces*
○ *Sporothrix*
○ *Aspergillus*
○ *Cryptococcus*
• Protozoa
○ *Toxoplasma*
○ *Leishmania*
• Metazoa
○ *Schistosoma*
• Spirochetes
○ *T. palladium*
○ *T. carateum*
○ *T. perunue*
• Mycobacteria
○ *M. tuberculosis*
○ *M. kansasii*
○ *M. leprae*
○ *M. marinum*
○ *M. avian*
○ BCG vaccine
• Bacteria
○ *Brucella*
○ *Yersinia*
**2. VASCULITIS**
• Wegener’s granulomatosis
• Necrotizing sarcoidal
• Churg–Strauss
• Broncho centric
• Polyarteritis nodosa
• Lymphomatoid
• Giant cell arteritis
• Systemic lupus erythematosus
• Beçhets disease
**3. IMMUNOLOGICAL ABERRATIONS**
• Crohn’s disease
• Orofacial granulomatosis
• Sarcoidosis
• Langerhan’s granulomatosis
• Hepatic granulomatous disease
• Primary biliary cirrhosis
• Blau’s syndrome
• Peyronie’s disease
• Immune complex disease
• Histiocytosis X
**4. CHEMICALS**
• Beryllium
• Zirconium
• Silica
• Starch
• Talc
**5. NEOPLASIA**
• Carcinoma
• Reticulosis
• Pinealoma
• Dysgerminoma
• Seminoma
• Reticulum cell sarcoma
• Malignant nasal granuloma
**6. MISCELLANEOUS INFECTIONS**
• Whipple’s disease
• Cat scratch
• Lymphogranuloma
• Kikuchi
• Buruli ulcer
**8. HYPERSENSITIVITY PNEUMONITIS**
• Farmers’ lung
• Bird fanciers’
• Suberosis (cork dust)
• Mushroom workers’
• Maple bark strippers’
• Bagassosis
• Paprika splitters’
• Spatlese lung
• Coffee bean
**10. IMMUNODEFICIENCIES**
• Chediak–Higashi
• Ataxia telangiectasia
• NBS
• RAG deficiency
• Artemis deficiency
• Jak3 deficiency
• HLA class I deficiency (TAP deficiency)
• Griscelli syndrome
• Common variable immunodeficiency
• Kabuki syndrome
• HIV (bacillary angiomatosis)
• CGD
• Hermansky–Pudlak
• Felty’s syndrome
• Common variable ID (CVID)
• Cartilage-hair hypoplasia SCID

### Infective granulomas

As the most frequent cause of granulomas, *Mycobacterium tuberculosis* has killed more people throughout history than any other infective disease (Lawn and Zumla, 2011). This suggests that tuberculous granulomas are not always efficient at eradicating this pathogen. Tuberculosis infections often manifest themselves in immunodeficiency states, suggesting that the innate immune system is key to maintaining effective granuloma formation and eradication of *M. tuberculosis*. Recent research shows that this intracellular pathogen can exploit the innate immune system in the early stages of granuloma formation to facilitate its persistence and potential systemic dissemination. On inhalation *M. tuberculosis* is rapidly phagocytosed by resident lung macrophages. They quickly replicate inside the macrophages and are transported to the surrounding tissues inside these cells. To try and prevent further dissemination and to ultimately exterminate the microorganism, granuloma formation is initiated.

The importance of the innate immune system in tuberculous granuloma formation is highlighted by the zebrafish model. Zebrafish larvae do not have an adaptive immune system, yet still form highly organized granulomas. Imaging studies inside zebrafish have shown macrophages arriving at a forming granuloma moving fluidly and rapidly within it (Davis et al., 2002). On encountering a dying macrophage, the incoming macrophage phagocytoses the dying cell and is subsequently infected with *M. tuberculosis*. The chemotaxis of macrophages into the granuloma is orchestrated by the *M. tuberculosis* RD-1 virulence locus, which encodes a type VII secretion system ESX-1 and its substrate ESAT-6. Strains lacking RD-1 have reduced macrophage ingress into what is a poorly formed granuloma and an attenuated infection. This is suggestive that *M. tuberculosis* utilizes granuloma formation as a protective mechanism to ensure its survival. TNFα was thought to play a central role in tuberculous granuloma formation as TNFα deficient mice showed poor granuloma formation. Studies in zebrafish have now shown this may not be the case as granulomas still form in the absence of TNFα, but are not maintained. Due to a lack of TNFα, macrophages are unable to effectively kill and digest the ingested Mycobacteria. The structure of the granuloma is lost in TNFα deficient animals when these overburdened macrophages die (Clay et al., 2008). The importance of TNFα in granuloma integrity is clearly illustrated in the reactivation of latent *M. tuberculosis* in humans undergoing anti-TNFα antibody therapy. Reactivation of infection is results from an impairment in bacterial containment due to the loss of a fully functional granuloma and reduced CD8^+^ T cell immunity during therapy (Miller and Ernst, 2009). Neutrophils also play a central role in the tuberculous granuloma with peak levels at 3 weeks after inoculation (Wolf et al., 2007). Failure of normal macrophage functioning in granuloma results in an increase of these cells in the granuloma. The consequence of impaired macrophage microbiocidal activity is the onset of necrosis which attracts neutrophils into the granuloma in response (Cooper et al., 1993; MacMicking et al., 2003). Increased neutrophil ingress has been shown to herald increased pathology in *M. tuberculosis* infections. Deficiency in caspase recruitment domain-containing protein 9 (CARD9) results in neutrophil-rich granulomas (Dorhoi et al., 2010; Redford et al., 2010). CARD9 mediates signals from so called pattern-recognition receptors such as toll-like receptors (TLRs), NOD-like receptors (NLRs), and dectin-1activate downstream pathways, including NF-κB. This subsequently induces the release of cytokines to stimulate/regulate the innate and adaptive immune responses. One such cytokine is the immunomodulator IL-10, essential for downregulation of IL-23 signaling and subsequent IL17 production. IL-17 is secreted by Th-17 and γδ T cells and leads to mass neutrophil influx into the granuloma (Lockhart et al., 2006). This is supported by the fact peak neutrophil influx occurs around day 21 and coincides with the activation of the adaptive immune response. In schistosome infection however, a Th-2 dominant response is mounted, absence of the Th-2 cytokine IL-4 results in increased hepatocyte damage, driven by excessive proinflammatory mediator secretion. This suggests the Th-2 response is protective for excessive immune stimulation and response to schistosome eggs (Pearce et al., 2004). Dorhoi et al. showed that CARD9 knockout mice failed to secrete IL-10 and produced neutrophilic granulomas due to increased IL-17 levels. These mice subsequently died from an aberrant excessive inflammatory response that resulted in the dissemination of the infection (Dorhoi et al., 2010). A role for CARD9 in the development of Crohn’s disease has also been highlighted through its identification in genome wide association studies (GWAS) (Franke et al., 2010).

### Complex disease of aberrant immune function

#### Sarcoidosis

Sarcoidosis is a systemic disease affecting multiple systems, but particularly barrier tissues such as the lungs, eyes, and skin (Rastogi et al., 2011). Current research has suggested that both environmental and genetic factors play a role in this complex disease. A GWAS conducted using 499 German individuals with sarcoidosis and 490 controls, detected an association to the ANXA11 (annexin 11) gene on chromosome 10q22.3 (Hofmann et al., 2008). This protein has been implicated in a number functions including vesicle trafficking and apoptosis (Gerke and Moss, 2002). It is thought that the prevention of propagation of granulomatous inflammation is controlled by apoptotic mechanisms of inflammatory cells. It facilitates calcium dependant interaction with ALG-2 a glycosyltransferase, which is necessary for caspase cell-induced death (Rao et al., 2004). It is therefore possible if annexin 11 regulated apoptotic mechanisms are affected because of this mutation, activated inflammatory cells survive and drive the granulomatous response further to cause wide spread disease.

High levels of TNFα and IL-12 in the serum of patients with the disease has been shown and also increased production of these cytokines by peripheral blood monocytes and alveolar macrophages (Prior et al., 1996). Macrophages are important defense cells in the lungs and are critical for initiating an inflammatory response on stimulation by inhaled microbes and toxins. This is facilitated through recognition of pathogen-associated molecular patterns (PAMPS) through pattern-recognition receptors such TLRs and NLRs. Rastogi et al. (2011) have shown that the high cytokine levels detected in patients with sarcoidosis is down to stimulation of alveolar macrophages with TLR4 and NOD1 agonists. This in turn leads to sustained p38 phosphorylation and cytokine production, particularly increased transcription of IL-12, TNFα, and IL-1 (Dong et al., 2002; Inoue et al., 2006). TNFα maintains the integrity and maintenance of the granuloma and limits the influx of inflammatory cells to the granuloma to prevent escalation of the inflammatory process.

Paradoxically, anti-TNFα induced sarcoidosis has also been reported in the literature, mainly affecting the lungs, parotid, and skin (Massara et al., 2010). After withdrawal of anti-TNFα therapy the condition resolves, it is thought that the cytokine imbalance experienced on prolonged TNFα blockade precludes the disease. Corticosteroid therapy has also been shown to be detrimental in recent onset disease (Reich, 2003, 2012).

Dendritic cells play a central role in granuloma formation by directly recruiting immune cells via TNFα secretion and indirectly through the activation of T cells via presentation of antigen in the surrounding lymph nodes. Patients with sarcoidosis have shown to present with a deficit in delayed type hypersensitivity reactions through impairment of dendritic cell functioning (Mathew et al., 2008). The same group also showed that sarcoid patients have comparable levels of circulating dendritic cells to that of healthy controls, but these dendritic cells display anergy to microbial challenge, despite the presence of upregulatied costimulatory and maturation markers (Mathew et al., 2008). This dysfunction is mild and therefore does not predispose these individuals to severe microbial infections as seen with those with primary immunodeficiencies. The same group also demonstrated a correlation between the degree of dendritic cell dysfunction and severity of pulmonary disease.

It has also been suggested that Tuberculosis and sarcoidosis are different spectrums of the same disease (Gupta et al., 2012). The histological and clinical similarities of both diseases could point to the fact that sarcoidosis is immune hypersensitivity to mycobacterial antigens, whilst in patients with tuberculosis; an underactive immune process.

#### Primary biliary cirrhosis

Activation of the innate immune system in this organ specific autoimmune disorder is thought to be key in the early stages of this disease as seen by eosinophilic infiltrations and elevated IgM (Berg, 2011). Biliary epithelial cells express a number of pattern-recognition receptors and are the first line of defense against foreign invaders in the liver. They are repeatedly exposed to PAMPS and through TLR and NLR signaling eventually undergo apoptosis. It is hypothesized that increased activation of apoptosis within these cells is via loss or down regulation of Bcl-2 and this in turn plays a major role in the disease process (Garchon et al., 1994). Poor clearance of these apoptotic and damaged cells by macrophages leads to their persistence and subsequent stimulation of the adaptive immune response and the tissue damage and granuloma formation that ensues, which is thought to be the primary mechanism driving the disease process. Particularly, exposure of mitochondrial membrane proteins leads to production of antimitochondrial autoantibodies (AMAs) which are a feature of the disease. This can lead to development of concomitant autoimmune disorders (Watt et al., 2004). The balance between bile duct proliferation and damage is the hallmark of this disease process which affects biliary epithelial cells.

### Granuloma formation in immunodeficiency states

A number of immunodeficiency disorders present with granulomatous inflammation, including hereditary immunodeficiency states such as common variable immunodeficiency and chronic granulomatous disease (CGD). Diseases which present with granulomas such as Crohn’s disease have also recently been shown to have an element of immunodeficiency (Rahman et al., 2008).

#### Crohn’s disease

One of the most common causes of granuloma formation in the gastrointestinal tract is the inflammatory bowel disorder; Crohn’s disease. Granulomas in Crohn’s patients can occur anywhere along the gastrointestinal tract from the mouth to the rectum. A complex interplay between the microbial contents, estimated to be 10^14^ bacteria, the epithelium and immune components drives this disease process. The gastrointestinal tract has a multi-layered defense system which consists of physical barriers, anti-bacterial molecules, and a range of leukocytes. Epithelial goblet cells secrete mucins, which act as a barrier to invading bacteria, whilst paneth cells secrete defensins which are primitive microbicidal peptides. Innate immune cells such as macrophages and dendritic cells are present in the lamina propria for immunosurveillance. It is though that a breakdown in this complex homeostasis between the gut microflora and host defense mechanisms is the precipitating cause to Crohn’s (Marks and Segal, 2008). A major hypothesis centers on the impaired handing and clearance of luminal content which gains access into the bowel wall (Sewell et al., 2009). The precise mechanisms responsible for this impaired innate immune response are still not fully understood. Recent GWAS (Barrett et al., 2008; Franke et al., 2010) have identified over 70 genetic polymorphisms associated with Crohn’s and this number is expected to increase over the next few years (Lees et al., 2011). Many of these loci have been linked to genes involved in bacterial recognition, innate immunity, and autophagy. Interestingly a large proportion of the genes identified are also associated with other immune conditions illustrating common pathological processes in quite different phenotypes (Lees et al., 2011).

Genome wide association studies have identified several genes associated with autophagy (*ATG16L1*, *IRGM*, *LRRK2*, *ULK1*, and *NOD2*) (Levine and Deretic, 2007). *NOD2* the most strongly associated gene with Crohn’s disease is responsible for pro-inflammatory cytokine release and autophagy induction (Hugot et al., 2001; Ogura et al., 2001; Travassos et al., 2010). Expressed predominantly by macrophages, NOD2 recruits another Crohn’s associated protein ATG16L1 to the cell membrane and acts as a sensor for bacterial peptidoglycan smallest subunit *N*-acetylmuramyl-L-alanyl-d-isoglutamine (MDP) and activating autophagy machinery (Torok et al., 2009). Numerous genetic studies have identified three *NOD2* polymorphisms rs2066844 (R702W), rs2066845 (G908R), and rs2066847 (1007fs) as being associated with increased susceptibility for Crohn’s disease, common to all three is a loss in MDP sensitivity and an inability to activated downstream targets, thus inducing an immunodeficiency in carriers (Burton et al., 2007; Franke et al., 2010). ATG16L is expressed in the epithelial cells of the intestine, in leukocytes, and the spleen (Fujita et al., 2008). Studies have shown that mutation of *ATG16L* (T300A) has an association with the formation of ileal Crohn’s disease (Prescott et al., 2007; Sventoraityte et al., 2010).

The mechanism of autophagy within the gut and has a number of functions to maintain homeostasis. The first of these is intracellular defense against microorganisms via the process of xenophagy (Gardet and Xavier, 2012). This is thought to be important in the tight control of the innate immune response against commensals gut organisms. If this process is affected and bacterial handling impaired, this can lead to an inappropriate host inflammatory response against commensal organisms. Some of these genes (*IRGM*, *NOD2*, *LRRK2*) are also associated with infective granulomatous disorders, thus suggesting Crohn’s patients may have altered control of mycobacterial infections (Intemann et al., 2009; Kumar et al., 2010). Autophagy proteins have also been shown to be involved in the regulation of the inflammasome through defects in mitochondrial autophagy leading to increase reactive oxygen species and loss of regulation of inflammasome pathways, particularly the pro-IL-1β pathway. Plantinga et al. (2011) demonstrated that mutation in ATG16L results in monocytes with increased IL1β secretion on stimulation with MDP. Dendritic cells also rely on autophagy for antigen presentation (Schmid et al., 2007). Recent evidence has suggested that defects in autophagy proteins may destabilize the immune cross talk between dendritic cells and T cells leading to an increased inflammatory response (Wildenberg et al., 2012). It can be assumed that this could add to the collateral damage caused by the immune system within the gut. The last function of autophagy is maintenance of secretory granules in paneth cells, which have a role in innate immune defense in the gut. NOD2 and ATG16L are expressed by paneth cells, and through NLR and TLR signaling pathways these cells release a cocktail of antimicrobial proteins, including lysozyme, but principally they secrete alpha defensins. These hydrophobic peptides are pore forming units that disrupt bacterial cell membrane leading to lysis and death. The 1007fs mutation in *NOD2* has been proven to decrease defensin expression and this is supported by the observation that patients with NOD2 mutations have lower defensins titers in ileostomy fluid compared to controls (Wehkamp et al., 2005; Van Limbergen et al., 2007). Lower levels of these proteins could facilitate entry of bacteria into the epithelial cells and a breach in the mucosal integrity. The T300A mutation in ATG16L also results in a decrease in secretory granule number within paneth cells. This loss of the mucosal barrier is the first step in the suggested multistep pathogenesis of Crohn’s (Marks and Segal, 2008). The next step involves reduction in bacterial killing capacity and clearance by neutrophils (Figure 1B).

Neutrophils play a central role in bacterial clearance from the gut and in the etiopathogenesis of Crohn’s. The importance of neutrophils in gut immunity becomes clear when you consider monogenic diseases that result in neutrophil dysfunction and the high incidence in bowel inflammation in these patients. These diseases are covered in more detail in the following section and a recent review (Rahman et al., 2008). A profound defect in the recruitment of neutrophils into tissues after stimulation with heat killed *Escherichia coli* and the subsequent failure to adequately clear this bacterial challenge has been identified in patients with Crohn’s disease (Smith et al., 2009). The neutrophils themselves function normally, but impaired cytokine secretion from macrophages results in their failure to accumulate in adequate numbers in the tissues (Marks et al., 2006; Smith et al., 2009). The consequence of this delayed recruitment results in bacteria remaining in the tissue, macrophages engulf the remaining bacteria and fecal matter inciting an inflammatory response culminating in granuloma formation. The defect is within the macrophage, specifically the vesicular trafficking and secretion of cytokines. Gene transcription and synthesis of TNFα was found to be normal in macrophages of Crohn’s disease patients, but secreted levels were grossly attenuated as a result of defective intracellular trafficking (Smith et al., 2009).

IL23R is another gene with several SNPs associated with Crohn’s disease (Duerr et al., 2006) as well as members of the IL23 pathway; Jak2 and STAT3 (Barrett et al., 2008). Secreted by activated dendritic cells, macrophages and monocytes, IL23 induces the developed of TH17cells and induces IL1, IL6, and TNFα from macrophages and monocytes. Primary immunodeficiencies characterized by mutations in IL23 pathway genes also present with granulomatous inflammation (see Table 2).

**Table 2:** **Immunodeficiency disorders that present with granulomatous inflammation**.

Disease	Gene	Inheritance	Granuloma location	Reference
Hermansky–Pudlak syndrome	*HPS-1*, *HPS-3*, 10q23.1-23.3	Autosomal recessive	GI tract	Schinella et al. (1980)
Chronic granulomatous disease	*NCF1*, *NCF2*, *CYBA*, and *CYBB*	X-linked or autosomal recessive	Liver, mouth, skin, lungs, urinary tract, GI tract	Segal et al. (1978)
Chediak–Higashi disease	*CHS1* (*LYST*), 1q42-43	Autosomal recessive	GI tract	Ishii et al. (1987)
RAG deficiency	*RAG1 RAG2* genes	Autosomal recessive	Cutaneous, mucous membranes, and internal organs	Schuetz et al. (2008)
Artemis deficiency (SCID-Athabascan)	*DCLRE1C*	Autosomal recessive	Cutaneous	IJspeert et al. (2011)
Janus kinase 3 (Jak3) deficiency	*ILR2G*	Autosomal recessive form of severe combined immunodeficiency (SCID)	Cutaneous	Gregoriou et al. (2008)
Griscelli syndrome	*RAB27a*	Autosomal recessive	Cutaneous	Eyer et al. (1998)
Common variable immunodeficiency (CVID)	*ICOS*, *TNFRSF13B* (encoding TACI), *TNFRSF13C* (encoding BAFF-R), and *CD19*.	Autosomal recessive and dominant forms	Lung, liver, skin, heart, eyes, gastrointestinal tract, and splenic granulomas	Ardeniz and Cunningham-Rundles (2009)
Ataxia telangiectasia	*A-T* mutated gene (11q22-23)	Autosomal recessive	Cutaneous granulomas	Chiam et al. (2011)
Nijmegen breakage syndrome (NBS)	*nbs1(8q21)*	Autosomal recessive	Cutaneous	Yoo et al. (2008)
TAP deficiency/bare lymphocyte syndrome type 1 group 3 (BLS)	*tap1 tap 2* genes	Autosomal recessive	Necrotizing cutaneous, gut and lung granulomas	Moins-Teisserenc et al. (1999), Zimmer et al. (2005)
Wiskott–Aldrich syndrome	WAS gene, on the X-chromosome (Xp11.23-p11.22	X-linked recessive	Cutaneous	Sebire et al. (2003)
Hyper IgM	CD40L gene long arm of the X-chromosome (Xq26-27.2)	X-linked recessive (autosomal recessive and dominant forms)	Cutaneous	Gallerani et al. (2004)

All these disorders have defects with the innate immune response and form granulomas in various tissues throughout the body.

The association of genes in autophagy regulation, IL-23 pathway, and defects in bacterial handling lends itself to the formation of the hypothesis that Crohn’s results from a defect in the digestion and removal of bacteria from the tissue. The remaining bacteria are walled off in the tissue and a granuloma forms to restrict dissemination of any infective agents. The granuloma then secretes pro-inflammatory cytokines and presents antigens to the adaptive immune system resulting in T cell activation. This response drives a chronic inflammatory condition which is the hallmark of Crohn’s disease and the focus of the majority of current therapeutic agents. Future drug design could target the defective bacterial clearance by boosting macrophage function and subsequently reducing granuloma formation which may prove advantageous.

#### Monogenic disorders of neutrophil function

Although neutrophil defects do not appear to be the primary cause of Crohn’s, disorders which result in low neutrophil numbers such as cyclical neutropenia, disorders of bacterial digestion such as CGD and disorders of impaired vesicle trafficking and expulsion (Chediak–Higashi and Hermansky–Pudlak syndromes), can present with a Crohn’s-like gut. This includes granuloma formation in the lamina propria and ulceration. Approximately half of all CGD patients develop bowel inflammation which is indistinguishable from Crohn’s disease (Marks et al., 2009). These observations support the important role of neutrophils in healthy gut maintenance and a reduction of functioning in granuloma formation.

Chronic granulomatous disease affects primarily males and is a rare congenital disease that is either X-linked or autosomal recessive (Holland, 2010). This disorder is characterized by a defect in the superoxide generating NADPH oxidase system in neutrophils which is necessary for killing microorganisms by respiratory burst. The pathognomic feature of CGD is recurrent infections, particularly by catalase positive bacteria and fungi (Martire et al., 2008). This impaired bacterial killing leads to granuloma formation in a variety of organs including the liver, gut, skin, and mouth (Levine et al., 2005). Defects of the proteins which are responsible for governing the respiratory burst to generate reactive oxygen species have been identified as the cause of this condition (Dinauer et al., 1987; Volpp et al., 1988; Clark et al., 1989). An unbalance in the expression of innate immune receptors on neutrophils has also been shown to have a causal role in CGD. Neutrophils in CGD patients show reduced expression of TLR5, TLR9, and CD18, reduced TLR5 levels resulting in reduced activation by bacterial flagella. Lowered CD18 expression results in impaired killing of *Staphylococcus aureus*. Both TLR5 and TLR9 expression levels have been shown to correlate with the severity of the disease, higher expression seen in patients with higher residual NADPH oxidase and a less severe phenotype (Rieber et al., 2012). Many CGD patients suffer from concomitant autoinflammatory conditions such as systemic lupus erythematosis and pulmonary fibrosis, potentially suggesting an uncontrolled inflammatory response tipping toward hyperinflammation (Winkelstein et al., 2000; Rieber et al., 2012). Upregulation of several pro-inflammatory genes has been identified by microarray analysis on activation of TLR2 or TLR4, which is independent of NADPH oxidase activity (Kobayashi et al., 2004; Bylund et al., 2007). In addition to the neutrophil the NADPH oxidase system is utilized by the macrophage and defective immune responses are also evident in CGD patients (Rahman et al., 2009). Impaired macrophage function is also evident in CGD, with attenuated pro-inflammatory cytokine and increased IL-10 secretion following bacterial stimulation. The role this plays in the development of granulomatous inflammation is still unclear.

### Autoinflammatory states

Blau syndrome is another immune disease which features granuloma formation. This rare autosomal dominant disorder can be characterized by granulomatous polyarthritis, uveitis, macropapular skin lesions containing granulomas and cranial neuropathies. Crohn’s-like disease occurs in 30% of patients (Geha et al., 2007). A missense mutation in *NOD2* drives this disease resulting in a gain of function and periodic episodes of fever and inflammation classic of this disorder (Fritz et al., 2006). This is a direct opposite of the loss of function seen in NOD2 associated with Crohn’s disease, although both conditions result in granuloma formation. Monocytes derived from patients with Blau syndrome after stimulation with macrophage colony-stimulating factor had a greater ability to form multinucleate giant cells compared to healthy controls (Yasui et al., 2010). Macrophages also displayed increased adherence and expression of inter-cellular adhesion molecule-1 (ICAM1), which could suggest an increase in ability for macrophage fusion and giant cell generation.

Blau syndrome granulomas are immunohistochemically distinct from those of Sarcoidosis or Crohn’s. A thick halo of lymphocytes, particularly Th17 cells surrounds the macrophages (Rose et al., 2011). Another observation in Blau syndrome granulomas is the phagocytosis of lymphocytes which can be found with their cytoplasms intact within the multinucleate giant cells of the granuloma. The gain of function of NOD2 and its role in autophagy could explain this phenomenon. Granulomas in patients with Crohn’s disease there is an absence of this halo of lymphocytes and an increase in neutrophils and sclerosis in the surrounding tissue (Janssen et al., 2012).

### Summary

Our understanding of the complex mechanisms that underlie granuloma formation and maintenance is growing, particularly the role of the innate immune system. Genetic studies have identified that many diseases of immune aberration and granuloma formation share common genetic backgrounds (Lees et al., 2011). Although, many of these diseases have multiple causal mutations associated with the disease process. Understanding the genetic overlap between these diseases is key to elucidating common pathways and mechanisms for the disease process in this specialized inflammatory response.

The key to finding new treatment strategies to combat granulomatous disorders lies with understanding the genetic and immunological background to immunodeficiency diseases that result in granuloma formation. Insight into the complex immune signaling pathways which drive these diseases will hopefully in turn highlight appropriate targets for novel therapeutic therapies. The fact that granuloma formation is associated with immunodeficiency states and also can be initiated in some diseases by immunomodulatory drugs highlights the need for caution in prescribing immunosuppressants to treat granulomatous diseases.

## Conflict of Interest Statement

The authors declare that the research was conducted in the absence of any commercial or financial relationships that could be construed as a potential conflict of interest.

## References

[B1] Ardeniz O. Cunningham-Rundles C. (2009). Granulomatous disease in common variable immunodeficiency. Clin. Immunol. 133, 198–207 10.1016/j.clim.2009.05.001 19716342 PMC2760682

[B2] Barrett J. C. Hansoul S. Nicolae D. L. Cho J. H. Duerr R. H. Rioux J. D. (2008). Genome-wide association defines more than 30 distinct susceptibility loci for Crohn’s disease. Nat. Genet. 40, 955–962 10.1038/ng.175 18587394 PMC2574810

[B3] Berg P. A. (2011). The role of the innate immune recognition system in the pathogenesis of primary biliary cirrhosis: a conceptual view. Liver Int. 31, 920–931 10.1111/j.1478-3231.2011.02457.x 21733082

[B4] Burton P. R. Clayton D. G. Cardon L. R. Craddock N. Deloukas P. Duncanson A. (2007). Genome-wide association study of 14,000 cases of seven common diseases and 3,000 shared controls. Nature 447, 661–678 10.1038/nature05911 17554300 PMC2719288

[B5] Bylund J. MacDonald K. L. Brown K. L. Mydel P. Collins L. V. Hancock R. E. W. (2007). Enhanced inflammatory responses of chronic granulomatous disease leukocytes involve ROS-independent activation of NF-kappa B. Eur. J. Immunol. 37, 1087–1096 10.1002/eji.200636651 17330823

[B6] Chiam L. Y. T. Verhagen M. M. M. Haraldsson A. Wulffraat N. Driessen G. J. Netea M. G. (2011). Cutaneous granulomas in ataxia telangiectasia and other primary immunodeficiencies: reflection of inappropriate immune regulation? Dermatology 223, 13–19 10.1159/000330335 21876338

[B7] Clark R. A. Malech H. L. Gallin J. I. Nunoi H. Volpp B. D. Pearson D. W. (1989). Genetic-variants of chronic granulomatous-disease – prevalence of deficiencies of 2 cytosolic components of the NADPH oxidase system. N. Engl. J. Med. 321, 647–652 10.1056/NEJM198909073211005 2770793

[B8] Clay H. Volkman H. E. Ramakrishnan L. (2008). Tumor necrosis factor signaling mediates resistance to mycobacteria by inhibiting bacterial growth and macrophage death. Immunity 29, 283–294 10.1016/j.immuni.2008.06.011 18691913 PMC3136176

[B9] Cooper A. M. Dalton D. K. Stewart T. A. Griffin J. P. Russell D. G. Orme I. M. (1993). Disseminated tuberculosis in interferon-gamma gene-disrupted mice. J. Exp. Med. 178, 2243–2247 10.1084/jem.178.6.2243 8245795 PMC2191280

[B10] Davis J. M. Clay H. Lewis J. L. Ghori N. Herbomel P. Ramakrishnan L. (2002). Real-time visualization of mycobacterium-macrophage interactions leading to initiation of granuloma formation in zebrafish embryos. Immunity 17, 693–702 10.1016/S1074-7613(02)00475-2 12479816

[B11] Dinauer M. C. Orkin S. H. Brown R. Jesaitis A. J. Parkos C. A. (1987). The glycoprotein encoded by the X-linked chronic granulomatous-disease locus is a component of the neutrophil cytochrome-B complex. Nature 327, 717–720 10.1038/327717a0 3600768

[B12] Dong C. Davis R. J. Flavell R. A. (2002). MAP kinases in the immune response. Annu. Rev. Immunol. 20, 55–72 10.1146/annurev.immunol.20.091301.131133 11861597

[B13] Dorhoi A. Desel C. Yeremeev V. Pradl L. Brinkmann V. Mollenkopf H. J. (2010). The adaptor molecule CARD9 is essential for tuberculosis control. J. Exp. Med. 207, 777–792 10.1084/jem.20090067 20351059 PMC2856020

[B14] Duerr R. H. Taylor K. D. Brant S. R. Rioux J. D. Silverberg M. S. Daly M. J. (2006). A genome-wide association study identifies IL23R as an inflammatory bowel disease gene. Science 314, 1461–1463 10.1126/science.1135245 17068223 PMC4410764

[B15] Egen J. G. Rothfuchs A. G. Feng C. G. Winter N. Sher A. Germain R. N. (2008). Macrophage and T cell dynamics during the development and disintegration of mycobacterial granulomas. Immunity 28, 271–284 10.1016/j.immuni.2007.12.010 18261937 PMC2390753

[B16] Eyer D. Petiau P. Finck S. Heid E. Grosshans E. Lutz P. (1998). Cutaneous granulomatous lesions in a patient with Griscelli syndrome. Annales de Dermatologie et de Venereologie 125, 727–728 9835967

[B17] Franke A. McGovern D. P. B. Barrett J. C. Wang K. Radford-Smith G. L. Ahmad T. (2010). Genome-wide meta-analysis increases to 71 the number of confirmed Crohn’s disease susceptibility loci. Nat. Genet. 42, 1118 10.1038/ng.553 21102463 PMC3299551

[B18] Fritz J. H. Ferrero R. L. Philpott D. J. Girardin S. E. (2006). Nod-like proteins in immunity, inflammation and disease. Nat. Immunol. 7, 1250–1257 10.1038/ni1412 17110941

[B19] Fujita N. Itoh T. Omori H. Fukuda M. Noda T. Yoshimori T. (2008). The Atg16L complex specifies the site of LC3 lipidation for membrane biogenesis in autophagy. Mol. Biol. Cell 19, 2092–2100 10.1091/mbc.E08-03-0289 18321988 PMC2366860

[B20] Gallerani I. Innocenti D. D. Coronella G. Berti S. Amato L. Moretti S. (2004). Cutaneous sarcoid-like granulomas in a patient with X-linked hyper-IgM syndrome. Pediatr. Dermatol. 21, 39–43 10.1111/j.0736-8046.2004.21107.x 14871324

[B21] Garchon H. J. Luan J. J. Eloy L. Bedossa P. Bach J. F. (1994). Genetic-analysis of immune dysfunction in non-obese diabetic (NOD) mice – mapping of a susceptibility locus close to the Bcl-2 gene correlates with increased resistance of NOD T-cells to apoptosis induction. Eur. J. Immunol. 24, 380–384 10.1002/eji.1830240217 8299687

[B22] Gardet A. Xavier R. J. (2012). Common alleles that influence autophagy and the risk for inflammatory bowel disease. Curr. Opin. Immunol. 24, 522–529 10.1016/j.coi.2012.08.001 23041451

[B23] Geha R. S. Notarangelo L. D. Casanova J. L. Chapel H. Conley M. E. Fischer A. (2007). Primary immunodeficiency diseases: an update from the International Union of Immunological Societies Primary Immunodeficiency Diseases Classification Committee. J. Allergy Clin. Immunol. 120, 776–794 10.1016/j.jaci.2007.08.053 17952897 PMC2601718

[B24] Gerke V. Moss S. E. (2002). Annexins: from structure to function. Physiol. Rev. 82, 331–371 11917092 10.1152/physrev.00030.2001

[B25] Gregoriou S. Trimis G. Charissi C. Kalogeromitros D. Stefanaki K. Rigopoulos D. (2008). Cutaneous granulomas with predominantly CD8(+) lymphocytic infiltrate in a child with severe combined immunodeficiency. J. Cutan. Med. Surg. 12, 246–248 18845095 10.2310/7750.2008.07061

[B26] Gupta D. Agarwal R. Aggarwal A. N. Jindal S. K. (2012). Sarcoidosis and tuberculosis: the same disease with different manifestations or similar manifestations of different disorders. Curr. Opin. Pulm. Med. 18, 506–516 10.1097/MCP.0b013e3283560809 22759770

[B27] Hofmann S. Franke A. Fischer A. Jacobs G. Nothnagel M. Gaede K. I. (2008). Genome-wide association study identifies ANXA11 as a new susceptibility locus for sarcoidosis. Nat. Genet. 40, 1103–1106 10.1038/ng.f.217 19165924

[B28] Holland S. M. (2010). Chronic granulomatous disease. Clin. Rev. Allergy Immunol. 38, 3–10 10.1007/s12016-009-8136-z 19504359

[B29] Hugot J. P. Chamaillard M. Zouali H. Lesage S. Cezard J. P. Belaiche J. (2001). Association of NOD2 leucine-rich repeat variants with susceptibility to Crohn’s disease. Nature 411, 599–603 10.1038/35079107 11385576

[B30] IJspeert H. Lankester A. C. van den Berg J. M. Wiegant W. van Zelm M. C. (2011). Artemis splice defects cause atypical SCID and can be restored in vitro by an antisense oligonucleotide. Genes Immun. 12, 434–444 10.1038/gene.2011.16 21390052

[B31] Inoue T. Boyle D. L. Corr M. Hammaker D. Davis R. J. Flavell R. A. (2006). Mitogen-activated protein kinase kinase 3 is a pivotal pathway regulating p38 activation in inflammatory arthritis. Proc. Natl. Acad. Sci. U.S.A. 103, 5484–5489 10.1073/pnas.0509188103 16567640 PMC1459381

[B32] Intemann C. D. Thye T. Niemann S. Browne E. N. L. Chinbuah M. A. Enimil A. (2009). Autophagy gene variant IRGM-261T contributes to protection from tuberculosis caused by *Mycobacterium tuberculosis* but not by *M. africanum* strains. PLoS Pathog. 5:e1000577 10.1371/journal.ppat.1000577 19750224 PMC2735778

[B33] Ishii E. Matui T. Iida M. Inamitu T. Ueda K. (1987). Chediak-Higashi-syndrome with intestinal complication - report of a case. J. Clin. Gastroenterol. 9, 556–558 10.1097/00004836-198710000-00015 3680907

[B34] James D. G. (2000). A clinicopathological classification of granulomatous disorders. Postgrad. Med. J. 76, 457–465 10.1136/pmj.76.895.301 10908370 PMC1741697

[B35] Janssen C. E. I. Rose C. D. De Hertogh G. Martin T. M. Meunier B. B. Cimaz R. (2012). Morphologic and immunohistochemical characterization of granulomas in the nucleotide oligomerization domain 2-related disorders Blau syndrome and Crohn disease. J. Allergy Clin. Immunol. 129, 1076–1084 10.1016/j.jaci.2012.02.004 22464675

[B36] Kobayashi S. D. Voyich J. M. Braughton K. R. Whitney A. R. Nauseef W. M. Malech H. L. (2004). Gene expression profiling provides insight into the pathophysiology of chronic granulomatous disease. J. Immunol. 172, 636–643 14688376 10.4049/jimmunol.172.1.636

[B37] Kumar D. Nath L. Kamal M. A. Varshney A. Jain A. Singh S. (2010). Genome-wide analysis of the host intracellular network that regulates survival of *Mycobacterium tuberculosis*. Cell 140, 731–743 10.1016/j.cell.2010.02.012 20211141

[B38] Lawn S. D. Zumla A. I. (2011). Tuberculosis. Lancet 378, 57–72 10.1016/S0140-6736(10)62173-3 21420161

[B39] Lees C. W. Barrett J. C. Parkes M. Satsangi J. (2011). New IBD genetics: common pathways with other diseases. Gut 60, 1739–1753 10.1136/gut.2009.199679 21300624

[B40] Levine B. Deretic V. (2007). Unveiling the roles of autophagy in innate and adaptive immunity. Nat. Rev. Immunol. 7, 767–777 10.1038/nri2161 17767194 PMC7097190

[B41] Levine S. Smith V. V. Malone M. Sebire N. J. (2005). Histopathological features of chronic granulomatous disease (CGD) in childhood. Histopathology 47, 508–516 10.1111/j.1365-2559.2005.02258.x 16241999

[B42] Lockhart E. Green A. M. Flynn J. L. (2006). IL-17 production is dominated by gamma delta T cells rather than CD4 T cells during *Mycobacterium tuberculosis* infection. J. Immunol. 177, 4662–4669 16982905 10.4049/jimmunol.177.7.4662

[B43] MacMicking J. D. Taylor G. A. McKinney J. D. (2003). Immune control of tuberculosis by IFN-gamma-inducible LRG-47. Science 302, 654–659 10.1126/science.1088063 14576437

[B44] Marks D. J. B. Harbord M. W. N. MacAllister R. Rahman F. Z. Young J. Al-Lazikani B. (2006). Defective acute inflammation in Crohn’s disease: a clinical investigation. Lancet 367, 668–678 10.1016/S0140-6736(06)68265-2 16503465

[B45] Marks D. J. B. Miyagi K. Rahman F. Z. Novelli M. Bloom S. L. Segal A. W. (2009). Inflammatory bowel disease in CGD reproduces the clinicopathological features of Crohn’s disease. Am. J. Gastroenterol. 104, 117–124 10.1038/ajg.2009.492_5 19098859

[B46] Marks D. J. B. Segal A. W. (2008). Innate immunity in inflammatory bowel disease: a disease hypothesis. J. Pathol. 214, 260–266 10.1002/path.2291 18161747 PMC2635948

[B47] Martire B. Rondelli R. Soresina A. Pignata C. Broccoletti T. Finocchi A. (2008). Clinical features, long-term follow-up and outcome of a large cohort of patients with chronic granulomatous disease: an Italian multicenter study. Clin. Immunol. 126, 155–164 10.1016/j.clim.2007.09.008 18037347

[B48] Massara A. Cavazzini L. La Corte R. Trotta F. (2010). Sarcoidosis appearing during anti-tumor necrosis factor alpha therapy: a new “class effect” paradoxical phenomenon. Two case reports and literature review. Semin. Arthritis Rheum. 39, 313–319 10.1016/j.semarthrit.2008.11.003 19147181

[B49] Mathew S. Bauer K. L. Fischoeder A. Bhardwaj N. Oliver S. J. (2008). The anergic state in sarcoidosis is associated with diminished dendritic cell function. J. Immunol. 181, 746–755 18566441 10.4049/jimmunol.181.1.746 PMC2593870

[B50] Miller E. A. Ernst J. D. (2009). Anti-TNFα immunotherapy and tuberculosis reactivation: another mechanism revealed. J. Clin. Invest. 119, 1079–1082 10.1172/JCI39143 19422095 PMC2673853

[B51] Moins-Teisserenc H. T. Gadola S. D. Cella M. Dunbar P. R. Exley A. Blake N. (1999). Association of a syndrome resembling Wegener’s granulomatosis with low surface expression of HLA class-I molecules. Lancet 354, 1598–1603 10.1016/S0140-6736(99)04206-3 10560675

[B52] Ogura Y. Bonen D. K. Inohara N. Nicolae D. L. Chen F. F. Ramos R. (2001). A frameshift mutation in NOD2 associated with susceptibility to Crohn’s disease. Nature 411, 603–606 10.1038/35079114 11385577

[B53] Pearce E. J. Kane C. M. Sun J. Taylor J. J. Mckee A. S. Cervi L. (2004). Th2 response polarization during infection with the helminth parasite *Schistosoma mansoni*. Immunol. Rev. 201, 117–126 10.1111/j.0105-2896.2004.00187.x 15361236

[B54] Plantinga T. S. Crisan T. O. Oosting M. van de Veerdonk F. L. de Jong D. J. Philpott D. J. (2011). Crohn’s disease-associated ATG16L1 polymorphism modulates pro-inflammatory cytokine responses selectively upon activation of NOD2. Gut 60, 1229–1235 10.1136/gut.2010.228908 21406388

[B55] Prescott N. J. Fisher S. A. Franke A. Hampe J. Onnie C. M. Soars D. (2007). A nonsynonymous SNP in ATG16L1 predisposes to ileal Crohn’s disease and is independent of CARD15 and 1BD5. Gastroenterology 132, 1665–1671 10.1053/j.gastro.2007.03.034 17484864

[B56] Prior C. Knight R. A. Herold M. Ott G. Spiteri M. A. (1996). Pulmonary sarcoidosis: patterns of cytokine release in vitro. Eur. Respir. J. 9, 47–53 10.1183/09031936.96.09010047 8834333

[B57] Rahman F. Z. Hayee B. Chee R. Segal A. W. Smith A. M. (2009). Impaired macrophage function following bacterial stimulation in chronic granulomatous disease. Immunology 128, 253–259 10.1111/j.1365-2567.2009.03112.x 19740382 PMC2767315

[B58] Rahman F. Z. Marks D. J. B. Hayee B. H. Smith A. M. Bloom S. L. Segal A. W. (2008). Phagocyte dysfunction and inflammatory bowel disease. Inflamm. Bowel Dis. 14, 1443–1452 10.1002/ibd.20449 18421761

[B59] Ramakrishnan L. (2012). Revisiting the role of the granuloma in tuberculosis. Nat. Rev. Immunol. 12, 352–366 22517424 10.1038/nri3211

[B60] Rao R. V. Poksay K. S. Castro-Obregon S. Schilling B. Row R. H. del Rio G. (2004). Molecular components of a cell death pathway activated by endoplasmic reticulum stress. J. Biol. Chem. 279, 177–187 10.1074/jbc.M402096200 14561754

[B61] Rastogi R. Du W. J. Ju D. H. Pirockinaite G. Liu Y. S. Nunez G. (2011). Dysregulation of p38 and MKP-1 in response to NOD1/TLR4 stimulation in sarcoid bronchoalveolar cells. Am. J. Respir. Crit. Care Med. 183, 500–510 10.1164/rccm.201005-0792OC 20851927 PMC5450927

[B62] Redford P. S. Boonstra A. Read S. Pitt J. Graham C. Stavropoulos E. (2010). Enhanced protection to *Mycobacterium tuberculosis* infection in IL-10-deficient mice is accompanied by early and enhanced Th1 responses in the lung. Eur. J. Immunol. 40, 2200–2210 10.1002/eji.201040433 20518032 PMC3378704

[B63] Reich J. M. (2003). Adverse long-term effect of corticosteroid therapy in recent-onset sarcoidosis. Sarcoidosis Vasc. Diffuse Lung Dis. 20, 227–234 14620167

[B64] Reich J. M. (2012). On the nature of sarcoidosis. Eur. J. Intern. Med. 23, 105–109 10.1016/j.ejim.2011.09.011 22284237

[B65] Rieber N. Hector A. Kuijpers T. Roos D. Hartl D. (2012). Current concepts of hyperinflammation in chronic granulomatous disease. Clin. Dev. Immunol. 2012:252460 10.1155/2012/252460 21808651 PMC3144705

[B66] Rose C. D. Martin T. M. Wouters C. H. (2011). Blau syndrome revisited. Curr. Opin. Rheumatol. 23, 411–418 10.1097/BOR.0b013e328349c430 21788900

[B67] Savill J. Fadok V. (2000). Corpse clearance defines the meaning of cell death. Nature 407, 784–788 10.1038/35037722 11048729

[B68] Schappi M. G. Deffert C. Fiette L. Gavazzi G. Herrmann F. R. Belli D. C. (2008). Branched fungal beta-glucan causes hyperinflammation and necrosis in phagocyte NADPH oxidase-deficient mice. J. Pathol. 214, 434–444 10.1002/path.2298 18098349

[B69] Schinella R. A. Greco M. A. Cobert B. L. Denmark L. W. Cox R. P. (1980). Hermansky-Pudlak Syndrome with Granulomatous Colitis. Ann. Intern. Med. 92, 20–23 10.7326/0003-4819-92-1-20 7350869

[B70] Schmid D. Pypaert M. Munz C. (2007). Antigen-loading compartments for major histocompatibility complex class II molecules continuously receive input from autophagosomes. Immunity 26, 79–92 10.1016/j.immuni.2006.10.018 17182262 PMC1805710

[B71] Schuetz C. Huck K. Gudowius S. Megahed M. Feyen O. Hubner B. (2008). An immunodeficiency disease with RAG mutations and granulomas. N. Engl. J. Med. 358, 2030–2038 10.1056/NEJMoa073966 18463379

[B72] Sebire N. J. Haselden S. Malone M. Davies E. G. Ramsay A. D. (2003). Isolated EBV lymphoproliferative disease in a child with Wiskott-Aldrich syndrome manifesting as cutaneous lymphomatoid granulomatosis and responsive to anti-CD20 immunotherapy. J. Clin. Pathol. 56, 555–557 10.1136/jcp.56.7.555 12835306 PMC1769998

[B73] Segal A. W. (2005). How neutrophils kill microbes. Annu. Rev. Immunol. 23, 197–223 10.1146/annurev.immunol.23.021704.115653 15771570 PMC2092448

[B74] Segal A. W. Webster D. Jones O. T. G. Allison A. C. (1978). Absence of a newly described cytochrome-B from neutrophils of patients with chronic granulomatous disease. Lancet 2, 446–449 10.1016/S0140-6736(78)91445-9 79807

[B75] Sewell G. W. Marks D. J. B. Segal A. W. (2009). The immunopathogenesis of Crohn’s disease: a three-stage model. Curr. Opin. Immunol. 21, 506–513 10.1016/j.coi.2009.06.003 19665880 PMC4529487

[B76] Smith A. M. Rahman F. Z. Hayee B. Graham S. J. Marks D. J. B. Sewell G. W. (2009). Disordered macrophage cytokine secretion underlies impaired acute inflammation and bacterial clearance in Crohn’s disease. J. Exp. Med. 206, 1883–1897 10.1084/jem.20091233090209c 19652016 PMC2737162

[B77] Sventoraityte J. Zvirbliene A. Franke A. Kwiatkowski R. Kiudelis G. Kupcinskas L. (2010). NOD2, IL23R and ATG16L1 polymorphisms in Lithuanian patients with inflammatory bowel disease. World J. Gastroenterol. 16, 359–364 10.3748/wjg.v16.i3.359 20082483 PMC2807958

[B78] Taylor R. C. Cullen S. P. Martin S. J. (2008). Apoptosis: controlled demolition at the cellular level. Nat. Rev. Mol. Cell Biol. 9, 231–241 10.1038/nrg2311 18073771

[B79] Torok P. Glas J. Endres I. Tonenchi L. Teshome M. Y. Wetzke M. (2009). Epistasis between Toll-like receptor-9 polymorphisms and variants in NOD2 and IL23R modulates susceptibility to Crohn’s disease. Am. J. Gastroenterol. 104, 1723–1733 10.1038/ajg.2009.184 19455129

[B80] Travassos L. H. Carneiro L. A. M. Ramjeet M. Hussey S. Kim Y. G. Magalhaes J. G. (2010). Nod1 and Nod2 direct autophagy by recruiting ATG16L1 to the plasma membrane at the site of bacterial entry. Nat. Immunol. 11, 55–62 10.1038/ni.1823 19898471

[B81] Van Limbergen J. Russell R. K. Nimmo E. R. Ho G. T. Arnott I. D. Wilson D. C. (2007). Genetics of the innate immune response in inflammatory bowel disease. Inflamm. Bowel Dis. 13, 338–355 10.1002/ibd.20096 17206667

[B82] Volpp B. D. Nauseef W. M. Clark R. A. (1988). Two cytosolic neutrophil oxidase components absent in autosomal chronic granulomatous-disease. Science 242, 1295–1297 10.1126/science.2848318 2848318

[B83] Watt F. E. James O. F. W. Jones D. E. J. (2004). Patterns of autoimmunity in primary biliary cirrhosis patients and their families: a population-based cohort study. QJM 97, 397–406 10.1093/qjmed/hch078 15208427

[B84] Wehkamp J. Salzman N. H. Porter E. Nuding S. Weichenthal M. Petras R. E. (2005). Reduced Paneth cell alpha-defensins in ileal Crohn’s disease. Proc. Natl. Acad. Sci. U.S.A. 102, 18129–18134 10.1073/pnas.0505256102 16330776 PMC1306791

[B85] Wildenberg M. E. Vos A. C. W. Wolfkamp S. C. S. Duijvestein M. Verhaar A. P. Te Velde A. A. (2012). Autophagy attenuates the adaptive immune response by destabilizing the immunologic synapse. Gastroenterology 142, 1493 10.1053/j.gastro.2012.02.034 22370477

[B86] Winkelstein J. A. Marino M. C. Johnston R. B. Boyle J. Curnutte J. Gallin J. I. (2000). Chronic granulomatous disease – report on a national registry of 368 patients. Medicine (Baltimore) 79, 155–169 10.1097/00005792-200005000-00003 10844935

[B87] Wolf A. J. Linas B. Trevejo-Nunez G. J. Kincaid E. Tamura T. Takatsu K. (2007). *Mycobacterium tuberculosis* infects dendritic cells with high frequency and impairs their function in vivo. J. Immunol. 179, 2509–2519 17675513 10.4049/jimmunol.179.4.2509

[B88] Yasui K. Yashiro M. Tsuge M. Manki A. Takemoto K. Yamamoto M. (2010). Thalidomide dramatically improves the symptoms of early-onset sarcoidosis/Blau syndrome its possible action and mechanism. Arthritis Rheum. 62, 250–257 10.1002/art.25035 20039400

[B89] Yoo J. Wolgamot G. Torgerson T. R. Sidbury R. (2008). Cutaneous noncaseating granulomas associated with Nijmegen breakage syndrome. Arch. Dermatol. 144, 418–419 10.1001/archderm.144.3.418 18347309

[B90] Zimmer J. Andres E. Donato L. Hanau D. Hentges F. de la Salle H. (2005). Clinical and immunological aspects of HLA class I deficiency. Qjm. 98, 719–727 10.1093/qjmed/hci112 16087697

